# Molecular Target Identification of Gossypol Against Cervical Cancer Based on Target Fishing Technology

**DOI:** 10.3390/pharmaceutics17070861

**Published:** 2025-06-30

**Authors:** Jinyan Li, Rayisa Asat, Wenying Li, Parwen Parhat, Yue Ma, Yinglan Ma, Min Li

**Affiliations:** 1 College of Pharmacy, Xinjiang Medical University, Urumqi 830011, China; jinyanli1015@163.com (J.L.); larisa1218@163.com (R.A.); wenyingli599@163.com (W.L.); 18371980618@163.com (P.P.); mayue9132@163.com (Y.M.); myl2443382113@163.com (Y.M.); 2 State Key Laboratory of Pathogenesis, Prevention and Treatment of High Incidence Diseases in Central Asia, Clinical Medical Research Institute, The First Affiliated Hospital of Xinjiang Medical University, Urumqi 830011, China; 3 Xinjiang Key Laboratory of Natural Medicines Active Components and Drug Release Technology, Xinjiang Medical University, Urumqi 830011, China; 4 Xinjiang Key Laboratory of Biopharmaceuticals and Medical Devices, Xinjiang Medical University, Urumqi 830011, China

**Keywords:** gossypol, target fishing, cervical cancer, magnetic microspheres

## Abstract

**Objectives**: This study aims to investigate the impact of Gossypol on human cervical cancer cells and elucidate its mechanism of action to establish a foundation for further clinical investigations. **Methods**: Cell proliferation, migration, and invasion were evaluated through CCK−8, wound healing, and Transwell assays. Fe_3_O_4_-BP-Gossypol (Fe_3_O_4_@Gossypol) conjugates were synthesized by linking Fe_3_O_4_ with Gossypol using benzophenone crosslinking. Successful conjugation was confirmed through scanning electron microscopy (SEM), Fourier transform infrared spectroscopy (FT-IR), and ultraviolet–visible spectrophotometry (UV-Vis). Subsequent to co-incubation with HeLa cell lysates, Fe_3_O_4_@Gossypol complexes facilitated the magnetic enrichment and purification of target proteins, which were identified using high-resolution mass spectrometry (HR-MS). The identified targets underwent KEGG pathway and GO analyses, followed by molecular docking with Gossypol. HeLa cells were exposed to Gossypol at concentrations of 7.48, 14.96, and 29.92 μmol·L^−1^ for 48 h, and protein expression levels were quantified via Western blotting. **Results**: Gossypol notably suppressed cervical cancer cell proliferation, migration, and invasion. The integration of target fishing, network pharmacology, and molecular docking highlighted PIK3R2, MAPK1, and GRB2 as potential therapeutic targets. Western blot analysis revealed a dose-dependent reduction in PIK3R2, GRB2, and MAPK1 expression in Gossypol-treated groups compared to controls (*p* < 0.05). **Conclusions**: Gossypol may exhibit anti-cervical cancer effects by modulating the PI3K/AKT signaling pathway.

## 1. Introduction

Cervical cancer (CC) is a prevalent gynecological malignancy globally, ranking as the fourth most common cancer among women worldwide and the leading cancer in certain developing nations. It is the second most common cause of cancer-related deaths in women aged 20–39 [1,2]. Annually, there are over 500,000 new cases of cervical cancer, resulting in approximately 250,000 deaths globally, with developing countries bearing about 80% of this burden [3]. The primary histological type of cervical cancer is squamous cell carcinoma (SCC), originating from the malignant transformation of squamous epithelial cells at the cervical transformation zone, which is the junction between the stratified squamous epithelium of the exocervix and the mucin-secreting columnar epithelium of the endocervical canal. Adenocarcinomas constitute the majority of the remaining cases, with a small percentage consisting of rare histological variations [4]. While surgical removal is effective for early-stage cervical cancer, many patients are diagnosed at advanced stages, requiring treatment with radiotherapy and chemotherapy [5]. However, managing advanced cervical cancer is challenging due to high recurrence rates, metastasis, toxicities from standard chemoradiotherapy, and the development of treatment resistance [6]. Therefore, the development of novel therapeutic agents with improved targeting precision and reduced systemic toxicity is crucial to enhance clinical outcomes in cervical cancer treatment.

**Figure 1:**
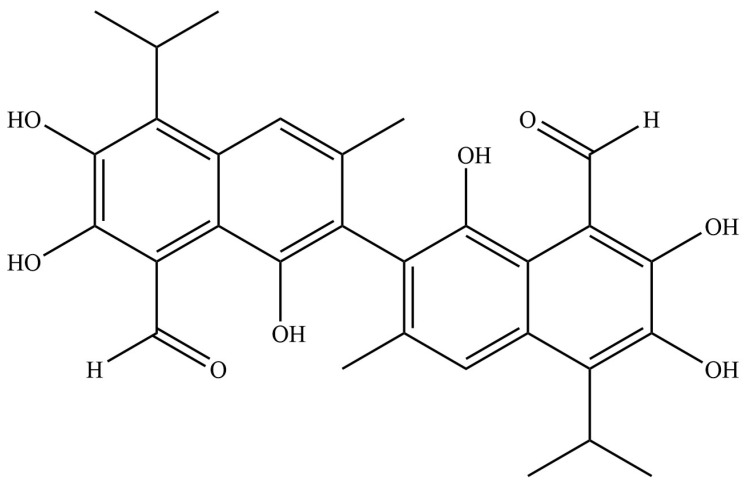
Structure of Gossypol.

Gossypol (C_30_H_30_O_8_) is a polyphenolic compound derived from cotton seeds, and it is chemically described as 2,2′-bis(8-formyl-1,6,7-trihydroxy-5-isopropyl-3-methylnaphthalene) (Figure 1). Initially developed as a male contraceptive, this dimeric sesquiterpene is now being explored as a potential anticancer agent. Recent research has focused on understanding its mechanisms for fighting tumors and its possible applications in clinical settings [7]. Preclinical studies have shown that Gossypol has various biological effects, such as anti-tumor properties, antioxidants, antiviral activity, broad-spectrum antimicrobial effects, and immune system modulation through the regulation of important signaling pathways [8]. Despite the known effectiveness of Gossypol in treating cervical cancer in experimental models, the specific molecular targets are not yet fully identified. Further research is needed to comprehensively characterize its mechanisms of action through integrated pharmacogenomic strategies and functional validation studies [9,10].

**Figure 2:**
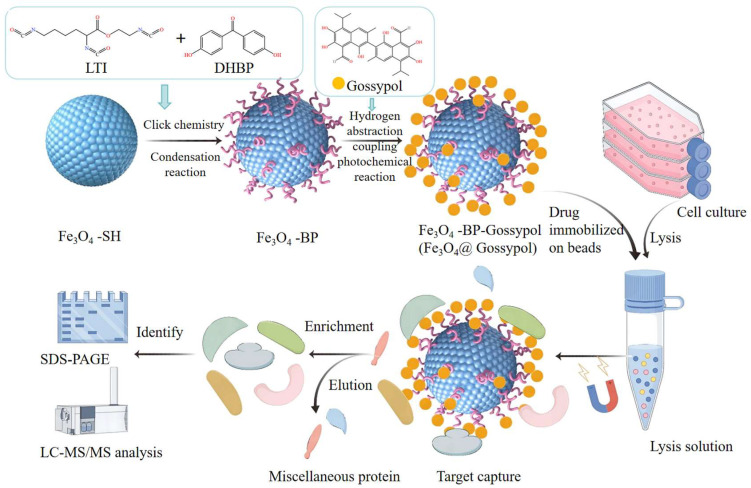
The synthesis of Fe_3_O_4_@Gossypol was achieved through click chemistry conjugation between isocyanate−functionalized thiol groups on Fe_3_O_4_ nanoparticles and 2,6−diisocyanatohexanoate (LTI), followed by condensation of the terminal isocyanate moieties of LTI with the hydroxyl groups of 4,4′−dihydroxybenzophenone (DHBP).

Drug targets are crucial focal points in pharmacological interventions, representing primary sites for drug-receptor interaction and forming the essential biological foundation for therapeutic effectiveness. Identifying targets not only aids in understanding the molecular mechanisms of drug actions but also allows for the predictive evaluation of off-target effects. This process is vital for optimizing the safety profiles of pharmacotherapies and improving the clinical translation in modern drug development pipelines [11]. Molecular “target fishing” technology, a widely accepted method in pharmacoproteomics, operates based on specific interactions between ligands and proteins. This approach involves the covalent attachment of drug molecules to functionalized solid-phase microbeads through controlled chemical modification. The resulting drug-conjugated matrix facilitates the selective capture and enrichment of target proteins through bioorthogonal binding. Subsequently, the proteins are isolated, purified, and structurally characterized using tandem mass spectrometry in conjunction with high-throughput proteomic profiling [12]. Based on this technology, researchers have successfully investigated the therapeutic targets of multiple natural bioactive components, which has significantly contributed to elucidating the pharmacological mechanisms of traditional Chinese medicine (TCM), accelerated the modernization process of TCM, and provided direct or indirect evidence for the identification and validation of natural product targets [13,14,15,16,17,18]. This research employed Fe_3_O_4_-SH nanoparticles to facilitate stepwise condensation polymerization between 2-isocyanatoethyl 2,6-diisocyanatohexanoate (LTI) and 4,4′-dihydroxybenzophenone (DHBP), yielding magnetically responsive microspheres grafted with light-sensitive benzophenone moieties. Following this synthesis, Gossypol was conjugated onto the magnetic microspheres’ surface, which were then incubated with lysed HeLa cells to selectively bind and isolate target proteins. The identified proteins were analyzed using high-resolution mass spectrometry (HRMS), and key therapeutic targets were screened and validated, followed by biological functional analysis (Figure 2). This study aims to elucidate the molecular mechanisms responsible for Gossypol’s anti-cervical cancer activity, with the goal of offering new pharmacological insights into its therapeutic effects. The results are anticipated to lay a scientific groundwork for the development of next-generation anti-cervical cancer agents characterized by precisely defined molecular targets and clearly elucidated mechanisms of action.

## 2. Materials and Methods

### 2.1. Chemicals and Reagents

The chemicals and reagents used in this research included the following: Fe_3_O_4_-SH particles (Junyijia Technology Co., Ltd., Tianjin, China); 2,3-dimercaptosuccinic acid (Aladdin Biochemical Technology Co., Ltd., Shanghai, China); 4,4′-dihydroxybenzophenone (Aladdin Biochemical Technology Co., Ltd., Shanghai, China); 1,8-diazabicyclo [5.4.0] undecane-7-ene (Aladdin Biochemical Technology Co., Ltd., Shanghai, China); 2-isocyanatoethyl 2,6-diisocyanatohexanoate (Rundong Chemical Co., Ltd., Quzhou, China); sodium hydroxide (Beilian Fine Chemicals Development Co., Ltd., Tianjin, China); methanol and ethanol (Xinbote Chemical Co., Ltd., Tianjin, China); acetone (Tiancheng Chemical Co., Ltd., Jiangsu, China); dimethyl sulfoxide (Sigma-Aldrich, St. Louis, MO, USA); Gossypol (purity ≥ 99.0%, Yirui Biotechnology Co., Ltd., Chengdu, China); DMEM high-glucose complete medium (containing 10% fetal bovine serum, Procell Life Technology Co., Ltd., Wuhan, China); 0.25% trypsin (Servicebio Biotechnology Co., Ltd., Wuhan, China); PBS buffer (Servicebio Biotechnology Co., Ltd., Wuhan, China); RIPA lysis buffer; and phosphatase inhibitor cocktail and PMSF protease inhibitor and BCA Protein Assay Kit (Solarbio, Beijing, China). The antibodies utilized included the following: anti-MAPK1 (Cell Signaling Technology, Inc., Danvers, MA, USA), anti-GRB2 and anti-PIK3R2 (Proteintech Group, Inc., Wuhan, China), and horseradish peroxidase (HRP)-conjugated secondary antibodies (Servicebio Biotechnology Co., Ltd., Wuhan, China).

### 2.2. Cell Culture

The human cervical cancer HeLa cell line was obtained from the Cell Bank of China Academy of Sciences (Beijing, China). Cells were cultured in a high-sugar DMEM medium supplemented with 10% fetal bovine serum (FBS), 100 U·L^−1^ of penicillin, and 100 µg·L^−1^ of streptomycin. Cell maintenance was carried out in a humidified incubator at 37 °C with 5% CO_2_ atmosphere [19,20]. To ensure optimal growth conditions, cells were subcultured every 48 h through standardized passaging procedures.

### 2.3. Cell Viability and IC50 Assay

Cell viability at 24 and 48 h was assessed via Cell Counting Kit-8 (CCK-8) (TargetMol, Boston, MA, USA). HeLa cells were cultured in 96-well plates and treated with varying concentrations of Gossypol (1, 2, 4, 6, 12, 18, 24, and 30 μmol·L^−1^) for 24 h and 48 h. Absorbance readings were taken at 450 nm with a microplate reader. The IC_50_ values for HeLa cells were determined using nonlinear regression analysis in GraphPad Prism (version 10.1.2, GraphPad Software, Boston, MA, USA) software based on the CCK-8 assay results [21].

### 2.4. Wound Healing Migration Assay

HeLa cells were seeded into 6-well plates and allowed to adhere. A linear scratch wound was created in the center of each well using a 200 μL pipette tip. The old culture medium was discarded, followed by three rinses with phosphate-buffered saline (PBS), and then, the cells were treated with different gradient concentrations of Gossypol (6, 12, and 18 μmol·L^−1^). Wound healing progression was documented at 0 h, 24 h, and 48 h post-treatment. Quantitative analyses of wound closure percentage were performed using ImageJ (version 1.54p, NIH, Bethesda, MD, USA) software [22].

### 2.5. Transwell Assay

In total, 200 microliters of serum-free DMEM cell suspension was inoculated into the upper chamber of the Transwell system, while 500 microliters of different concentrations (6, 12, and 18 μmol·L^−1^) of Gossypol was added to the lower chamber. The plates were incubated in a humid environment containing 5% carbon dioxide at 37 °C for 48 h. Subsequently, cells were fixed with 4% paraformaldehyde for 30 min and stained with 0.1% crystal violet solution for 30 min. Cells that migrated to the lower side of the membrane were visualized using an inverted microscope. The invasion rate was quantified by counting the number of cells penetrating the pores/basement membrane and expressed as a percentage relative to the control group [23].

### 2.6. Fe_3_O_4_@Gossypol

#### 2.6.1. Preparation of DHBP-Functionalized Fe_3_O_4_ NPs

Graft polymerization was conducted by utilizing the surface thiol (-SH) groups of Fe_3_O_4_−SH particles. Specifically, 10 mg of Fe_3_O_4_-SH particles was dispersed in 34 mL of acetone within a 100 mL three-neck flask with stirring at 200 rpm. To this suspension, L-lysine triisocyanate (LTI), 4,4′-dihydroxybenzophenone (DHBP), and the initiator 1,8-diazabicyclo [5.4.0] undec-7-ene (DBU) were sequentially added. The reaction proceeded at room temperature for 30 min before adding 2,3-dimercaptosuccinic acid (DMSA), and the reaction continued for an additional 2.5 h. The resulting product was magnetically separated and washed five times with methanol to obtain polymer-functionalized magnetic particles (designated as Fe_3_O_4_-BP particles) incorporating LTI, DHBP, and DMSA. The purified Fe_3_O_4_-BP particles were finally dispersed in methanol for subsequent applications [13,24].

#### 2.6.2. Gossypol onto DHBP-Bound Fe_3_O_4_ NPs

**Figure 3:**
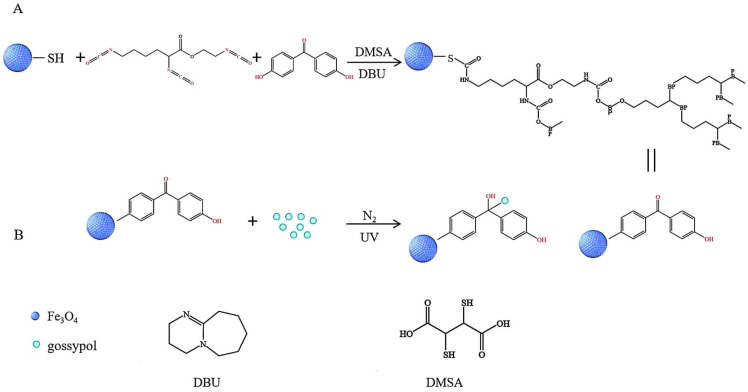
(**A**) Synthesis of photosensitive Fe_3_O_4_ magnetic particles via polymerization. (**B**) Formation of drug−conjugated magnetic particles (designated as Fe_3_O_4_@Gossypol) through the surface binding of Gossypol to Fe_3_O_4_−BP particles. The abbreviation BP in the Fe_3_O_4_@Gossypol structure denotes benzophenone.

The photochemical reaction was performed in a round-bottomed photoreactor, using -SH group on the surface of Fe_3_O_4_-SH (Figure 3). The reactor was filled with 20 mL of Gossypol methanol solution (0.25 mg·mL^−1^) and 5 mg of Fe_3_O_4_-BP particles. The mixture was purged with N_2_ for 30 min to establish an inert atmosphere, followed by UV irradiation (375 W mercury lamp; light intensity: 12.5 W·m^−2^; λ = 254 nm) for 1 h. This procedure yielded magnetic particles with surface-conjugated Gossypol, designated as Fe_3_O_4_-BP-Gossypol (Fe_3_O_4_@Gossypol), which were subsequently collected for further use [13,24].

### 2.7. Characterization of Magnetic Microspheres

#### 2.7.1. Field-Emission SEM (FE-SEM) Analysis

Particle morphology was examined using a field-emission scanning electron microscope (SU8100, Hitachi High-Technologies Corporation, Tokyo, Japan). The Fe_3_O_4_-SH magnetic particles and synthesized Fe_3_O_4_@Gossypol nanoparticles were immobilized on silicon wafers and imaged under FE-SEM at an acceleration voltage of 10 kV and a magnification of ×25,000 [25,26].

#### 2.7.2. Fourier Transform Infrared (FT-IR) Spectroscopic Study

Dried specimens of Fe_3_O_4_-SH, Fe_3_O_4_-BP, Gossypol, and Fe_3_O_4_@Gossypol (2 mg each) were thoroughly combined with 200 mg of potassium bromide (KBr) in an agate mortar and ground into fine powders. Each homogenized mixture was subsequently compressed into translucent pellets under a defined pressure using a hydraulic press. Fourier transform infrared (FT-IR) (Shimadzu, Kyoto, Japan) spectra were acquired in the range of 4000–400 cm^−1^ with a resolution of 4 cm^−1^ using KBr pellets as the background, with comparative analysis performed to identify structural variations among the samples [13,26].

#### 2.7.3. Ultraviolet-Visible (UV-Vis) Spectrophotometry Analysis

UV-vis spectra of Gossypol, Fe_3_O_4_-SH, Fe_3_O_4_-BP, and Fe_3_O_4_@Gossypol were measured from 200 to 800 nm on a Shimadzu UV-2700 spectrophotometer (Shimadzu, Kyoto, Japan). Additionally, the content of Gossypol conjugated to the magnetic particles was quantified via polymer degradation analysis and supernatant analysis [26].

### 2.8. Protein Extraction

HeLa cells were seeded in 6-well plates and cultured for 24 h. Subsequently, the cells were washed with phosphate-buffered saline (PBS) and lysed with a RIPA lysis buffer containing 1% phenylmethylsulfonyl fluoride (PMSF) and 2% phosphatase inhibitor cocktail. The lysate underwent an ice bath for half an hour before being centrifuged at 12,000 rpm for ten minutes at 4 °C, with the resulting supernatant preserved for subsequent analysis [27].

### 2.9. Enrichment of Target Proteins

Protein lysates were incubated with Fe_3_O_4_-SH (control group) and Fe_3_O_4_@Gossypol (conjugation group) at 4 °C overnight to enable the efficient capture of target proteins. Subsequently, the samples were rinsed thrice with phosphate-buffered saline (PBS) to remove unbound proteins.

### 2.10. LC-MS/MS Analysis of Target Proteins

A reaction buffer was added to the sample, followed by incubation at 95 °C for 10 min to achieve protein denaturation, reduction, and alkylation. The sample was centrifuged, and the supernatant was collected and diluted with an equal volume of ddH_2_O. Trypsin was introduced for protein breakdown, followed by overnight incubation at 37 °C with agitation. The digestion was terminated by adding trifluoroacetic acid (TFA) the next day. After centrifugation, the supernatant was desalted using a custom-made SDS desalting column.

All samples were analyzed on an UltiMate 3000 RSLCnano system (Thermo, Waltham, MA, USA) coupled online to a Q Exactive HF mass spectrometer through a Nanospray Flex ion source (Thermo, Waltham, MA, USA). Peptide samples were loaded via an autosampler onto a C18 trap column (75 µm × 2 cm, 3 µm, 100 Å pore size; Thermo Scientific, Waltham, MA, USA) and subsequently separated on a custom-made analytical column (75 µm × 25 cm, 1.9 µm, 100 Å pore size). A gradient was created with mobile phase A (0.1% formic acid, 3% DMSO, 97% H_2_O) and B (0.1% formic acid, 3% DMSO, 97% CAN), flowing at 300 nL·min^−1^. Information was gathered in the DDA (Data-Dependent Acquisition) mode. The MS1 full scan was configured with a resolution of 60,000 @ 200 m·z^−1^, a scan range of 350–1500 m⋅z^−1^, an AGC target of 3 × 10⁶; the max injection duration was capped at a rapid 30 milliseconds. To ensure purity, we established an isolation window for precursor ions at 1.4 Da, pinpointing the top 20 most potent ones for fragmentation. We utilized a High-Collision Dissociation (HCD) energy of 28% to achieve this. For MS2 scans, the parameters were configured as follows: resolution of 15,000 @ 200 m·z^−1^, AGC target of 1 × 10^5^, max injection duration of 50 milliseconds, and dynamic exclusion span of 30 s.

### 2.11. KEGG and GO Analyses

The differential analysis of the “hook fishing” protein between control and experimental groups was conducted through mass spectrometry and protein database comparisons. Subsequently, differentially expressed proteins were identified and subjected to KEGG and GO analyses using Metascape (https://metascape.org). The identified proteins were then cross-referenced with potential targets for cervical cancer treatment from the GeneCards database (https://www.genecards.org) to select key proteins. The 3D structure of the selected protein was obtained from the RCSB PDB database (https://www.rcsb.org) in pdb format, followed by dehydration and hydrogenation using AutoDockTools 1.5.6 software to generate pdbqt format files. The structure of Gossypol was retrieved from PubChem (https://pubchem.ncbi.nlm.nih.gov) and converted to pdbqt format using Open Babel 3.1.1. Molecular docking simulations were performed with AutoDockVina 1.2.3, and the model with the optimal binding energy was visualized using PyMOL 3.0.4 [28,29].

### 2.12. Western Blot

Cells were washed three times with PBS and lysed using a RIPA buffer containing protease and phosphatase inhibitors. The concentration of proteins was assessed with the BCA Protein Assay Kit. Subsequently, proteins were diluted with a loading buffer and denatured at 100 °C for 10 min at 100 °C; there were then stored at −20 °C. Samples were subjected to 10% SDS-PAGE gel electrophoresis and membrane transfer. Membranes were treated with 5% non-fat milk at room temperature for 2 h, followed by overnight incubation at 4 °C with gentle shaking in the presence of specific primary antibodies targeting MAPK1, GRB2, and PIK3R2. After three TBST washes, the samples were exposed to HRP-linked Goat Anti-Rabbit or HRP-linked Goat Anti-Mouse antibodies under ambient conditions for a duration of 60 min. After three TBST rinses, protein bands were detected with an ECL kit, using β-actin as the loading control. Protein band intensities were assessed via densitometric quantification using ImageJ software [27,30].

### 2.13. Statistical Analysis

All data are expressed as mean ± standard deviation (SD) and analyzed using Prism 10 (GraphPad Software). One-way analysis of variance (ANOVA) was performed for intergroup comparisons under the assumption of homogeneity of variance. For datasets violating this assumption (heteroscedasticity), the Kruskal–Wallis test (non-parametric rank-sum equivalent) was applied. Post hoc pairwise comparisons were made via the Student-Newman-Keuls (SNK) method. A threshold of *p* < 0.05 was defined as statistically significant for all analyses.

## 3. Results

### 3.1. Gossypol Inhibits Proliferation, Invasion, and Migration in HeLa Cells

**Figure 4:**
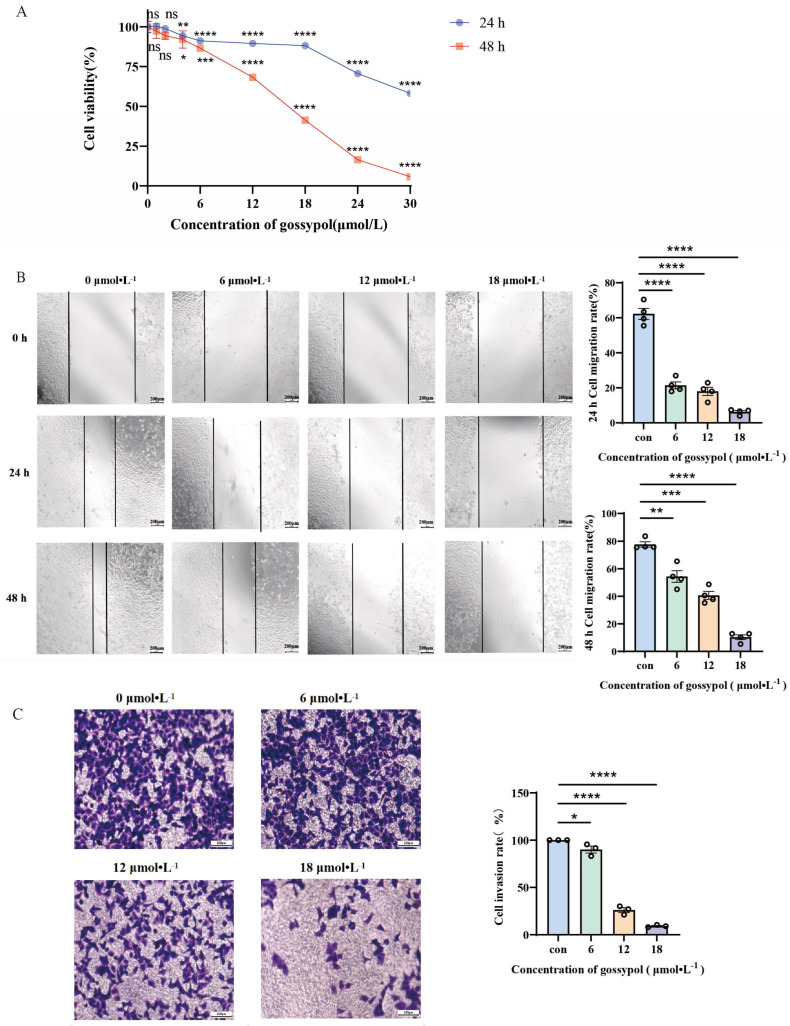
(**A**) The effect of Gossypol on HeLa cell viability was assessed using the CCK–8 assay. (**B**) Cell migration was analyzed via a wound healing assay. (**C**) Cell invasion was evaluated using the Transwell assay. Statistical significance is ns denotes non–statistically significant variations. * Significance level: *p* < 0.05. ** Significance level: *p* < 0.01. *** Significance level: *p* < 0.001. **** Significance level: *p* < 0.0001 when compared to the control, *n* = 3.

In order to examine the impact of Gossypol on HeLa cells, diverse dosages of the compound were applied to the cells. The CCK-8 assay results demonstrated that Gossypol significantly reduced the viability of human HeLa cells in a dose-dependent manner, with IC_50_ values of 36.41 μmol·L^−1^ and 14.96 μmol·L^−1^ at 24 h and 48 h (Figure 4A), respectively. Compared with cisplatin (IC_50_: 11.42 μmol·L^−1^ at 24 h, 6.57 μmol·L^−1^ at 48 h) (see Supplementary Figure S1), although gossypol showed a higher half inhibitory concentration, functional experiments confirmed that it significantly inhibited the movement of HeLa cells. Wound healing assays revealed that control group cells exhibited notable migratory capacity in the wound area, whereas Gossypol treatment at concentrations of 6, 12, and 18 μmol·L^−1^ significantly suppressed the cell migration rate after 48 h intervention (Figure 4B). Additionally, Transwell invasion assays indicated that the number of invasive cells in Gossypol–treated HeLa cells was markedly reduced compared to the control group (Figure 4C).

### 3.2. Conjugation Analysis of Drug Molecules on Magnetic Particle Surfaces

**Figure 5:**
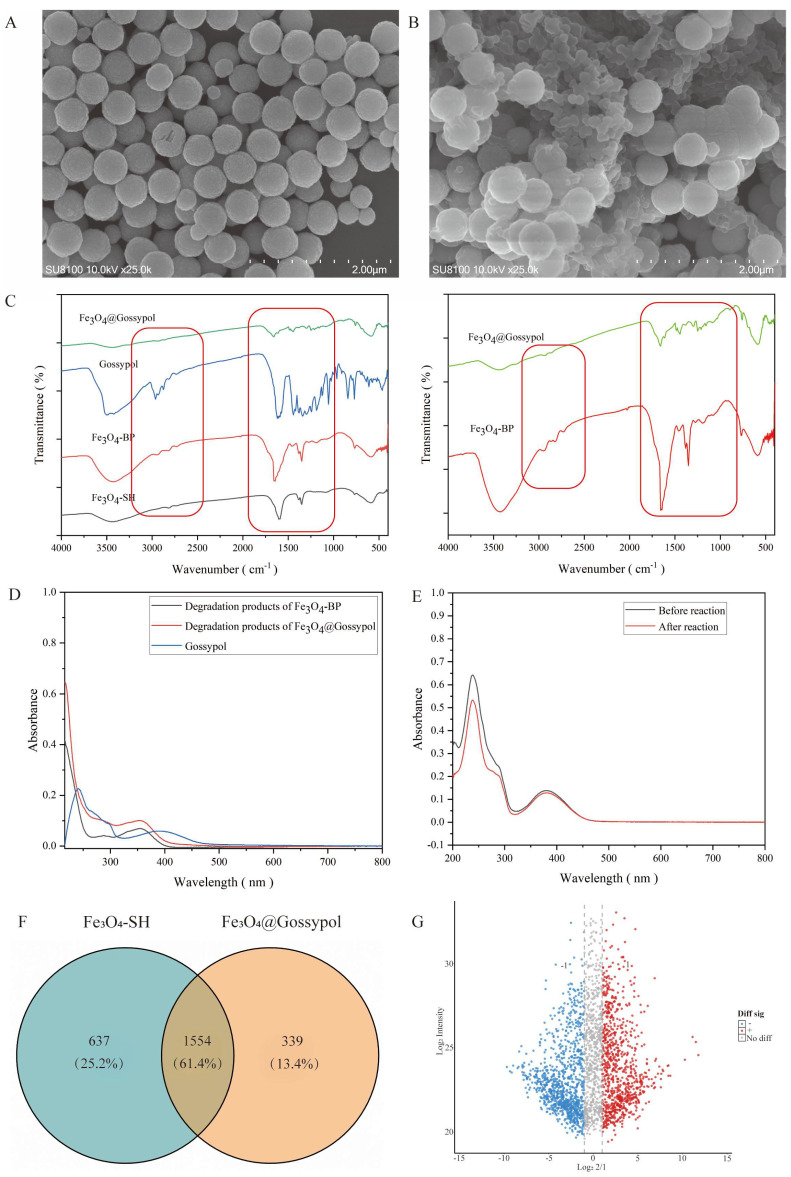
(**A**) SEM image of Fe_3_O_4_–SH. (**B**) SEM image of Fe_3_O_4_@Gossypol. (**C**) Comparative FT–IR spectra before and after Gossypol conjugation (The red boxes indicate the differences in the infrared spectra of different substances). (**D**) UV–Vis spectra comparison of Fe_3_O_4_−BP and Fe_3_O_4_@Gossypol following alkaline hydrolysis. (**E**) UV absorption spectra comparison of supernatants pre− and post−Gossypol conjugation. (**F**) Protein overlap diagram between Fe_3_O_4_−SH and Fe_3_O_4_@Gossypol. (**G**) Volcano plot. Symbols: − denotes significant downregulation, + denotes significant upregulation, and No diff indicates no significant difference.

Compared to Fe_3_O_4_–SH magnetic nanoparticles (Figure 5A), Fe_3_O_4_@Gossypol magnetic nanoparticles (Figure 5B) exhibited significant morphological alterations and a distinct core–shell coupling pattern. FT–IR analysis revealed a characteristic absorption peak at 590 cm^−1^ corresponding to the Fe-O-Fe bond. However, no prominent –SH absorption peak (expected at 2570 cm^−1^) was observed, likely due to its weak signal intensity. The analysis of Fe_3_O_4_–BP particle composition demonstrated a characteristic absorption peak at 1650 cm^−1^, attributed to the keto carbonyl group (–C=O) of the BP moiety, confirming the successful conjugation of DHBP on the magnetic nanoparticle’s surface. Separately, the methyl (–CH_3_) and methylene (–CH_2_–) groups exhibited asymmetric stretching at 2954 cm^−1^ and symmetric stretching at 2860 cm^−1^. Following Gossypol conjugation, the maximum absorbance of the keto carbonyl peak at 1650 cm^−1^ was markedly reduced compared to pre–conjugation spectra. The spectral similarity between Gossypol and Fe_3_O_4_@Gossypol further supports the occurrence of photochemical conjugation between the BP moiety and Gossypol (Figure 5C).

To further analyze drug molecules conjugated to the magnetic nanoparticle’s surface, a polymer degradation–based analytical method was employed. Specifically, alkaline hydrolysis was performed to remove surface-grafted polymers and conjugated Gossypol from the nanoparticles, followed by UV–Vis spectroscopic analysis to quantify Gossypol bound to the magnetic particle surface. The results showed that the BP moiety in Fe_3_O_4_–BP exhibited distinct absorption maxima occurring at 280–300 nm and approximately 350 nm, while free Gossypol displayed characteristic peaks at 240 nm and 320–480 nm. For Fe_3_O_4_@Gossypol, absorption peaks near 350 nm were observed, but no Gossypol-specific peaks were detected. Overlapping UV absorption bands between Fe_3_O_4_@Gossypol and the BP moiety (Figure 5D) precluded the reliable identification of surface–conjugated drug molecules. To address this, supernatant analysis was adopted. UV–Vis spectroscopy was applied to analyze the supernatants of Fe_3_O_4_–BP particles and Gossypol dispersions before and after light irradiation. By comparing changes in drug molecule concentrations (via standard calibration curves), the amount of drugs conjugated to the magnetic particle surface was calculated. The results demonstrated a significant decrease in Gossypol concentration in the supernatant post-irradiation (Figure 5E), unequivocally confirming successful Gossypol conjugation to the magnetic nanoparticles.

### 3.3. Gossypol Hook Fishing Proteomic Analysis Based on High-Resolution Mass Spectrometry

Fe_3_O_4_–SH magnetic beads detected 2191 proteins, while Fe_3_O_4_@Gossypol identified 1893 proteins, with 1554 overlapping proteins. Volcano plots were generated using the log_2_ (spectral count ratio) as the x-axis and the log_2_ (average spectral count) as the y–axis (Figure 5G). Differential protein screening thresholds were applied, with statistical significance annotated based on protein scatter distribution. The analysis of differential proteins between Fe_3_O_4_@Gossypol and Fe_3_O_4_–SH revealed 890 upregulated and 983 downregulated proteins. Additionally, 630 proteins showed no significant differential expression, while 27 proteins lacked quantifiable data. Among the upregulated proteins, 306 were unique to Fe_3_O_4_@Gossypol and were prioritized for further investigation (Table 1).

**Figure 6:**
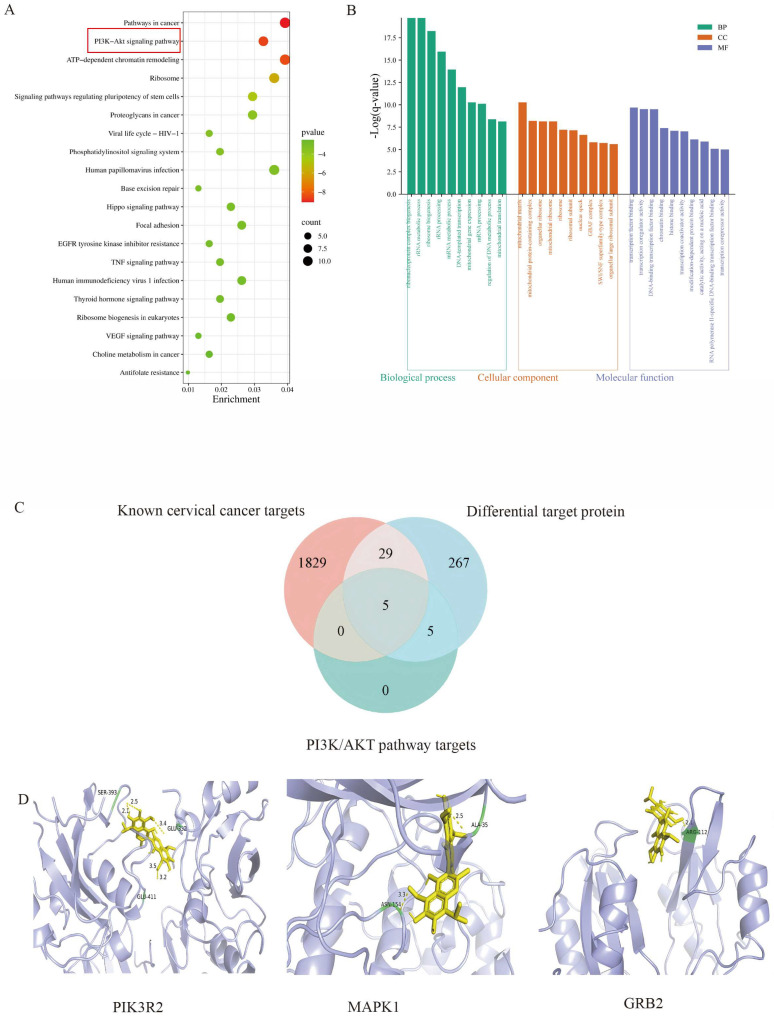
(**A**) KEGG pathway analysis. (**B**) Gene Ontology (GO) analysis. (**C**) Venn diagram illustrating the intersection of Fe_3_O_4_@Gossypol hook differential proteins, Gossypol−associated targets, and PI3K/AKT pathway components. (**D**) Molecular docking models of Gossypol with PIK3R2, GRB2, and MAPK1 proteins.

To better elucidate the biological roles of the identified proteins, the 306 differentially expressed targets were subjected to Gene Ontology (GO) functional analysis and Kyoto Encyclopedia of Genes and Genomes (KEGG) pathway enrichment analyses using Metascape (Figure 6A,B). KEGG pathway analysis revealed significant enrichment in the pathways closely associated with cervical carcinogenesis, including cancer pathways, phosphatidylinositol 3–kinase/protein kinase B (PI3K/AKT) signaling, ATP–dependent chromatin remodeling, EGFR tyrosine kinase inhibitor resistance, TNF signaling, and VEGF signaling.

The molecular docking analysis of the 306 differential proteins with known cervical cancer–related targets identified intersecting proteins within the PI3K/AKT signaling pathway: PIK3R2, GRB2, MAPK1, FGF2, and PRKCA (Table 2). The binding energies between Gossypol and these target proteins were all <−5 kJ·mol^−1^, demonstrating robust binding affinity and stability. Among these, PIK3R2, GRB2, and MAPK1 were prioritized for further mechanistic investigation.

PIK3R2, also known as p85β, serves as a regulatory subunit of phosphatidylinositol 3–kinase (PI3K) and functions as a proto–oncogene. It plays a pivotal role in regulating cellular growth, survival, proliferation, motility, and morphology by activating associated cascade reactions through the PI3K/AKT signaling pathway. Specifically, p85β promotes tumorigenesis by modulating PI3K activity and downstream AKT signaling [31]. Numerous studies have demonstrated the aberrant expression of PIK3R2 in various malignancies, which is closely associated with enhanced cellular proliferation and invasive capacity, as well as reduced apoptosis [32]. For instance, Cortés I et al. identified elevated p85β expression in breast and colorectal cancers, with increased expression levels strongly correlated with PI3K pathway activation and tumor progression [33]. Notably, p85β induces moderate PIP3 production at the plasma membrane, thereby augmenting cancer cell invasiveness. Similarly, PIK3R2 upregulation has been observed in cervical cancer, ultimately contributing to distant recurrence [34]. However, research in this area remains limited.

GRB2 functions as a pivotal intracellular adapter that is vital for cell division, featuring a central SH2 domain surrounded by two SH3 domains [35]. It coordinates accurate signaling pathways from membrane receptors to intracellular responses, a process involving signal transduction and gene expression. GRB2 plays a pivotal role across numerous physiological functions, amongst them modulating innate and adaptive immune responses, facilitating autophagy, aiding DNA restoration, and mediating necroptosis. Within the EGFR signaling axis, it acts as a molecular bridge between autophagy and programmed necrosis through interactions with RIPK1 and autophagy regulators SQSTM1 and BECN1. Notably, GRB2 protein overexpression has been documented in various malignancies, including cervical cancer, and is strongly associated with enhanced cancer cell motility, migration, and invasiveness [36]. This finding further underscores GRB2’s critical role in mediating cancer cell biological behaviors.

MAPK1, also known as ERK2, forms a pivotal element within the mitogen–activated protein kinase (MAPK) signaling pathway. Depending on cell type, the MAPK/ERK cascade mediates diverse biological functions through the regulation of transcription, translation, and cytoskeletal reorganization, including cellular growth, adhesion, survival, and differentiation. Furthermore, this signaling axis plays a crucial role in the initiation and modulation of meiosis, mitosis, and postmitotic functions in differentiated cells through the phosphorylation of multiple transcription factors. Notably, the aberrant activation of MAPK1 exhibits strong associations with various pathological conditions, particularly malignancies [37,38,39]. In multiple cancer types, frequent mutations in Ras genes drive the constitutive activation of the downstream MAPK signaling pathway, thereby promoting neoplastic cell proliferation. Consequently, MAPK1 not only governs fundamental cellular homeostasis but has also emerged as a potential therapeutic target for cancer treatment, with specific relevance to cervical carcinoma management [40].

### 3.4. Effects of Gossypol on MAPK1, PIK3R2, and GRB2 Protein Expression in HeLa Cells

**Figure 7:**
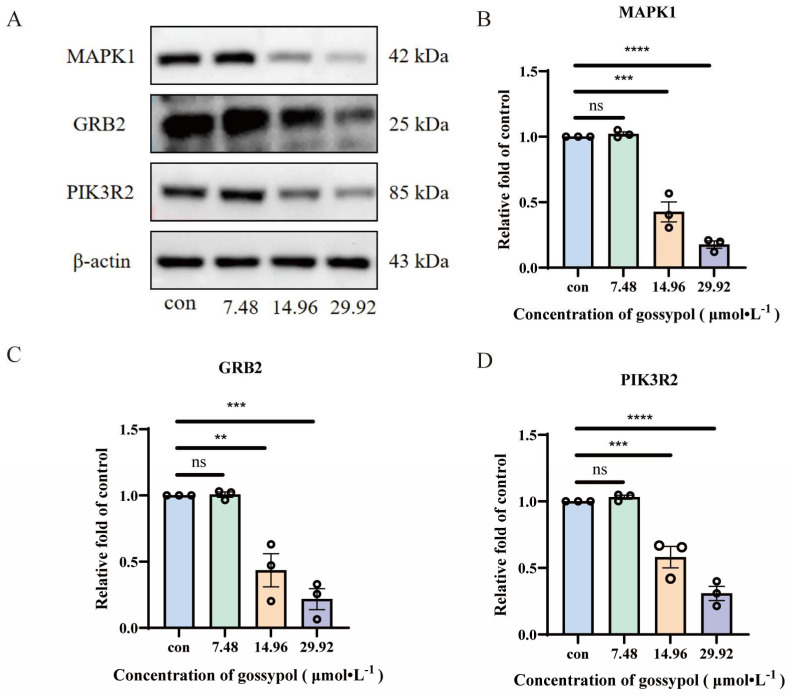
(**A**) Protein band pattern. (**B**) Effects of Gossypol on MAPK1 protein expression. (**C**) Effects of Gossypol on GRB2 protein expression. (**D**) Effects of Gossypol on PIK3R2 protein expression. Statistical significance is ns indicates no significant difference. ** *p* < 0.01, *** *p* < 0.001, and **** *p* < 0.0001 vs. Con, *n* = 3.

Gossypol treatment decreased the expression of MAPK1, PIK3R2, and GRB2 proteins with an increase in Gossypol dose (Figure 7A). In contrast to the blank group, the relative expression of MAPK1, PIK3R2, and GRB2 proteins could be significantly reduced by Gossypol in the medium– and high–concentration groups, all of which were statistically significant (*p* < 0.05) (Figure 7B–D).

## 4. Discussion

This investigation examined the impact of Gossypol on HeLa cell viability, invasion, and migration using the CCK-8 assay, Transwell assay, and wound healing experiment. The results indicated that Gossypol significantly reduced HeLa cell viability and inhibited both invasive and migratory capabilities, consistent with previous studies by Hsieh YS [9] and Li Y [10]. Magnetic nanoparticles were successfully synthesized with surface-conjugated photosensitive groups, allowing for the covalent immobilization of Gossypol onto solid-phase microspheres via a hydrogen-abstraction-coupling photochemical reaction facilitated by benzophenone (BP) moieties under ultraviolet (UV) irradiation. Subsequent exposure to HeLa cell lysate facilitated the capture of target proteins associated with cervical carcinogenesis. The functional analysis of these proteins revealed that Gossypol primarily acts through the PI3K/AKT signaling pathway and ATP-dependent chromatin remodeling. The PI3K/AKT pathway plays a crucial role in regulating cellular homeostasis, impacting processes such as growth, energy utilization, and longevity. Importantly, this pathway has been extensively linked to tumor progression, survival mechanisms, and resistance to therapy. Previous research has demonstrated that inhibiting the PI3K/AKT pathway effectively suppresses the proliferation, invasion, and migration of cervical cancer cells [23,41,42,43]. These findings underscore the importance of investigating the specific mechanisms and potential therapeutic implications of modulating the PI3K/AKT pathway in managing cervical cancer.

The subsequent analysis of the 306 identified targets, in conjunction with the PI3K/AKT signaling pathway-enriched proteins and established cervical cancer-associated targets, led to the identification of five potential candidate proteins. Molecular docking simulations were conducted to assess the binding affinity between Gossypol and these candidates, revealing binding energies below −5 kJ·mol^−1^, indicating robust binding affinity and thermodynamic stability. Three proteins, namely, PIK3R2, GRB2, and MAPK1, were prioritized for further investigation. PIK3R2 plays a crucial role in regulating cell growth, survival, proliferation, motility, and morphological dynamics, while GRB2 is involved in cancer cell mobility, migration, and invasion through growth factor signal transduction. Additionally, MAPK1 governs fundamental cellular processes such as adhesion, differentiation, and apoptosis. The observed anti-cervical cancer effects of Gossypol are likely attributed to its multi-target modulation, disrupting oncogenic signaling networks through coordinated interactions with these key proteins.

This study utilized bioinformatics functional analysis, molecular docking, and known cervical cancer-related targets to reveal that Gossypol’s therapeutic effects on cervical cancer primarily involve the PI3K/AKT signaling pathway. The analysis identified three key target proteins-PIK3R2, GRB2, and MAPK1-showing significant associations with Gossypol. It is important to note that while these findings highlight important targets, they do not represent the full scope of potential target proteins and pathways involved in Gossypol’s anti-cervical cancer mechanisms. The current analytical constraints limit the exploration of the complete range of target proteins and therapeutic pathways. Undiscovered proteins may play vital roles as targets, necessitating further validation through experimental investigations at the cellular and protein levels to fully elucidate the compound’s pharmacological network.

Moreover, the spatial conformation of Gossypol may undergo changes when conjugated to magnetic microspheres, potentially leading to distinctions between proteins bound to Fe_3_O_4_@Gossypol and those affected by free Gossypol in the cellular environment. While the impact of Fe_3_O_4_ conjugation on Gossypol conformation may be minimal, unsuccessful conjugation could be due to various factors that warrant further experimental exploration for clarification.

## 5. Conclusions

This study employed photoaffinity labeling technology to identify the protein profile targeted by Gossypol and elucidated its mechanism of action against cervical cancer through bioinformatic analyses. This research establishes a novel molecular-level therapeutic approach for treating cervical cancer. The results not only offer a clear direction for future mechanistic inquiries but also improve understanding of drug–target interactions in complex traditional Chinese medicine systems. This progress significantly advances herbal medicine research, with substantial scientific implications and translational potential. However, the current findings are confined to in vitro experiments and require validation through in vivo studies. Subsequent research will concentrate on systematic in vivo investigations to further confirm and enhance this research framework.

## Figures and Tables

**Table 1:** Statistical summary of differential proteins.

diff.sig	Fe_3_O_4_@Gossypol/Fe_3_O_4_-SH
Up count	890
Down count	983
No diff	630
NA	27

**Table 2:** Gossypol–protein molecular docking affinities.

No.	PDB ID	Protein Name	Binding Energy (kJ⋅mol^−1^)
1	8OM6	FGF2	−6.3
2	6ICG	GRB2	−6.9
3	7RNU	PIK3R2	−9.1
4	4DNL	PRKCA	−6.2
5	8AOJ	MAPK1	−7.3

## Data Availability

The raw data supporting the conclusions of this article will be made available by the authors upon request.

## Supplementary Materials

The following supporting information can be downloaded at https://www.mdpi.com/article/10.3390/pharmaceutics17070861/s1. Figure S1: Cytotoxicity of Cisplatin on HeLa cells. Cells were treated with Cisplatin at 0–32 µM for 24 h, 48 h, and cell viability was determined by CCK-8 assay and analyzed by GraphPad Prism software (10.1.2).
