# Supplementary material for: Molecular Target Identification of Gossypol Against Cervical Cancer Based on Target Fishing Technology
Source: Pharmaceutics. 2025 Jun 30;17(7):861. doi: 10.3390/pharmaceutics17070861 (PMC12298003; doi:10.3390/pharmaceutics17070861)
Supplement: Supplementary file 1 [file pharmaceutics-17-00861-s001.zip › pharmaceutics-3693900-supplementary.pdf]

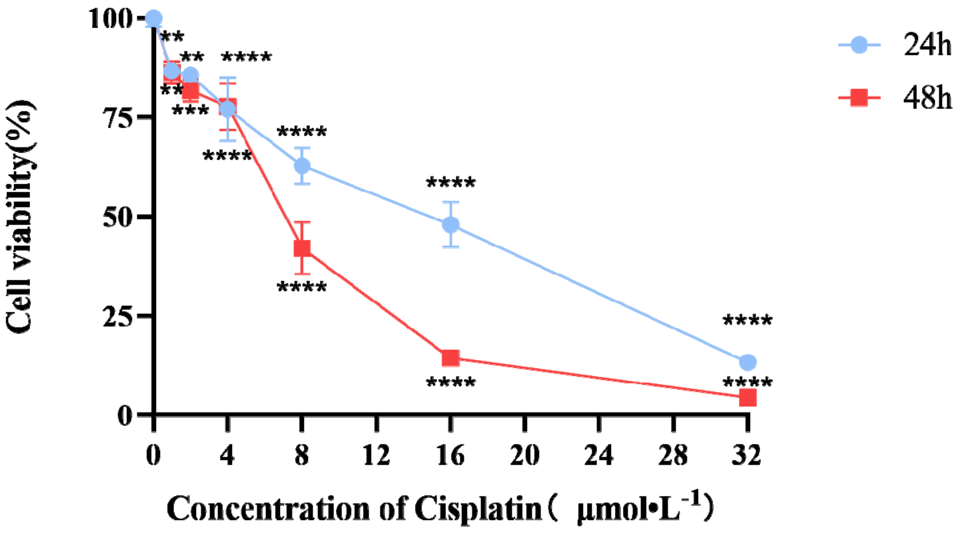

Figure S1. Cytotoxicity of Cisplatin on HeLa cells. Cells were treated with Cisplatin at 0–32 μM for 24 h, 48 h, and cell viability was determined by CCK−8 assay and analyzed by GraphPad Prism software (10.1.2).
